# Brain Changes in Responders vs. Non-Responders in Chronic Migraine: Markers of Disease Reversal

**DOI:** 10.3389/fnhum.2016.00497

**Published:** 2016-10-06

**Authors:** Catherine S. Hubbard, Lino Becerra, Jonathan H. Smith, Justin M. DeLange, Ryan M. Smith, David F. Black, Kirk M. Welker, Rami Burstein, Fred M. Cutrer, David Borsook

**Affiliations:** ^1^Center for Pain and the Brain, Department of Anesthesiology, Perioperative and Pain Medicine, Boston Children’s HospitalBoston, MA, USA; ^2^Department of Anaesthesia, Harvard Medical SchoolBoston, MA, USA; ^3^Department of Neurology, Mayo ClinicRochester, MN, USA; ^4^Department of Anaesthesia, Beth Israel Deaconess Medical CenterBoston, MA, USA

**Keywords:** headache, pain, preventative therapy, BOTOX^®^, migraine transformation, fMRI, gray matter, network connectivity

## Abstract

The aim of this study was to identify structural and functional brain changes that accompanied the transition from chronic (CM; ≥15 headache days/month) to episodic (EM; <15 headache days/month) migraine following prophylactic treatment with onabotulinumtoxinA (BoNT-A). Specifically, we examined whether CM patients responsive to prophylaxis (responders; *n* = 11), as evidenced by a reversal in disease status (defined by at least a 50% reduction in migraine frequency and <15 headache days/month), compared to CM patients whose migraine frequency remained unchanged (non-responders; *n* = 12), showed differences in cortical thickness using surface-based morphometry. We also investigated whether areas showing group differences in cortical thickness displayed altered resting-state functional connectivity (RS-FC) using seed-to-voxel analyses. Migraine characteristics measured across groups included disease duration, pain intensity and headache frequency. Patient reports of headache frequency over the 4 weeks prior to (pre-treatment) and following (post-treatment) prophylaxis were compared (post minus pre) and this measure served as the clinical endpoint that determined group assignment. All patients were scanned within 2 weeks of the post-treatment visit. Results revealed that responders showed significant cortical thickening in the right primary somatosensory cortex (SI) and anterior insula (aINS), and left superior temporal gyrus (STG) and pars opercularis (ParsOp) compared to non-responders. In addition, disease duration was negatively correlated with cortical thickness in fronto-parietal and temporo-occipital regions in responders but not non-responders, with the exception of the primary motor cortex (MI) that showed the opposite pattern; disease duration was positively associated with MI cortical thickness in responders versus non-responders. Our seed-based RS-FC analyses revealed anti-correlations between the SI seed and lateral occipital (LOC) and dorsomedial prefrontal cortices (DMPFC) in responders, whereas non-responders showed increased connectivity between the ParsOp seed and LOC. Overall, our findings revealed distinct morphometric and functional brain changes in CM patients that reverted to EM following prophylactic treatment compared to CM patients that showed no change in disease status. Elucidating the CNS changes involved in disease reversal may be critical to discovering interventions that prevent or slow the progression of CM. Such changes may aid in the evaluation of treatments as well as provide markers for disease “de-chronification”.

## Introduction

Migraine continues to be a common and debilitating condition, associated with significant economic, societal and personal burden. While the majority of migraineurs experience low to moderate episodic migraine (EM ≥15 headache days/month), in many patients this condition progresses in frequency to chronic migraine (CM), defined as ≥15 headache days/month (Headache Classification Subcommittee of the International Headache Society, 2004; Olesen et al., 2006). For example, 3% of individuals in the general population with infrequent episodic headache progress to CM each year (Lipton et al., 2015). Given a 1-year prevalence of EM in the US of nearly 12% (Lipton et al., 2007), these percentages translate into millions of patients at risk for progression or transformation. Undoubtedly, any intervention that could impede disease progression or reverse a chronic state to episodic would significantly impact the lives of patients. However, little is known about the neurobiology that mediates CM disease progression, or that accompanies disease reversal.

Migraine is not only a sensory problem during ictal periods (pain, photophobia, phonophobia), but there is accumulating evidence of morphological and functional brain changes during the interictal phase (Valfré et al., 2008; Maleki et al., 2012; Hubbard et al., 2014; Hodkinson et al., 2015; Chong et al., 2016a,b). Our group has reported that the migraine brain undergoes significant functional and structural plasticity with increased frequency of attacks (Maleki et al., 2011, 2012, 2013). For instance, differences in blood-oxygen-level dependent (BOLD) signal responses during painful heat stimulation have been observed in the anterior insula (aINS) and primary somatosensory cortex (SI) and were found to be associated with gray matter (GM) changes in high frequency vs. low frequency EM patients (Maleki et al., 2012). Because these alterations in migraineurs might represent markers of the disease state, we evaluated the structural and functional brain changes that accompanied improvements in migraine status in patients with CM that have reverted to EM as a result of prophylactic treatment (i.e., responders) compared to those patients that showed no change in disease status (i.e., non-responders).

OnabotulinumtoxinA (BoNT-A) is a neurotoxin reportedly efficacious as a prophylactic treatment for CM (Lipton et al., 2011; Frampton, 2012). In a series of randomized, double-blind, placebo-controlled studies, BoNT-A injected intramuscularly to facial and pericranial sites, reduced the number of headache days, average headache duration and severity of symptoms in patients with CM, with minimal adverse events (Diener et al., 2010; Dodick et al., 2010; Aurora et al., 2011; Lipton et al., 2016). Recent studies by Burstein et al. (2009) have provided compelling evidence that BoNT-A effects may occur by selective inhibition of meningeal C-fiber trigeminal afferents (high-threshold mechano-nociceptors) by interfering with TRPV1 and TRPA1 ligand receptor binding (Burstein et al., 2014; Zhang et al., 2016). While the response rate to BoNT-A itself is relatively small in randomized, placebo-controlled studies, differences in responders vs. non-responders have been reported based on initial phenotype. Specifically, so called “exploding” headache is resistant to BoNT-A injections and may differentiate the lack of effects of the drug on intracranial innervation in this group; in contrast, “imploding” headaches may be due to drug actions on extracranial trigeminal mechano-nociceptor afferents innervating the meninges (Jakubowski et al., 2006; Burstein et al., 2009). Thus, the migraine phenotype and by implication, the premorbid brain state, may predict treatment responders from treatment non-responders.

In this study, we hypothesized that CM patients who have reverted to EM after successful treatment intervention using BoNT-A would show distinct structural and functional brain changes that coincided with decreases in migraine headache frequency compared to CM patients unresponsive to treatment, and showed no change in attack frequency. It should be noted that the approach taken here does not rule out potential placebo responses (i.e., non-specific effects of the treatment intervention), or pre-treatment (premorbid) brain status that may have contributed to treatment/placebo efficacy and/or resistance. Whatever the processes involved, the treatment intervention produced two different groups for analysis—responders and non-responders. An avenue for future research may be to explore machine learning-based approaches (López et al., 2013) to predict BoNT-A treatment responsiveness and differentiate responders from non-responders using the brain regions identified in the present study.

## Materials and Methods

### Patient Selection, Study Design and Procedures

This study was approved by the Mayo Clinic Institutional Review Board and conducted in accordance with the Declaration of Helsinki. Prior to enrollment and commencement of all study-related procedures, participants gave their written informed consent. A total of 24 patients (18–59 years of age; mean age = 38.92 ± 12.75; 18 females) with a diagnosis of CM participated in this study. All patients were identified and recruited retrospectively through a search of the Mayo Headache Registry or from outpatient headache clinics. Patients were recruited to participate in this study if they had received prior BoNT-A injections for treatment of their CM and met all inclusionary and exclusionary criteria described below. A history and physical along with a full neurological examination, as part of patient’s routine clinical care, were performed by a board certified neurologist (F. Michael Cutrer, MD; Jonathan H. Smith, MD; Justin M. DeLange, DO; Ryan M. Smith, DO). Patients were deemed eligible for inclusion if they met diagnostic criteria for CM according to the International Classification of Headache Disorders-II (ICHD-II) guidelines at the initial clinical visit, prior to any treatment with BoNT-A (Headache Classification Subcommittee of the International Headache Society, 2004); diagnostic criteria included a history of recurring migraine headaches for at least the last 3 months and a headache frequency ≥15 days/month. Other inclusion criteria required that patients were between the ages of 18–60 years and right-handed. Patients were excluded from the study if they reported any current or previous history of significant peripheral or CNS disease, pregnancy or plans to become pregnant, claustrophobia, weight >235 lbs (MRI limit), significant alcohol consumption (ingestion of 5 or more glasses of alcohol per week; >40 oz./week), metal implants or devices considered ferromagnetic, a history of taking opioids for >6 months, and/or a history of psychiatric disorders based on standard clinical history.

At the initial clinical visit, prior to BoNT-A administration, demographic information and age of disease onset (defined as the approximate age patient first reported having migraine headaches) were assessed along with self-report ratings on the Patient Health Questionnaire (version 9; PHQ-9; Kroenke et al., 2001) and the Migraine Disability Assessment (MIDAS; Stewart et al., 2000) scale. The PHQ-9 and the MIDAS were administered to screen for depression and evaluate the impact of CM on daily functioning, respectively. In addition, disease severity including pain intensity (in the last 4 weeks on a 0–10 numerical rating scale [NRS]; 0 = no pain and 10 = worst pain imaginable) and migraine headache frequency (average number of headache days in the last 4 weeks) were also determined. Self-report and disease severity measures assessed at the initial clinical visit, prior to BoNT-A administration, served as a baseline (pre-treatment; pre-Tx) for comparison with the post-treatment (post-Tx) measures obtained during the follow-up visit. All patients were exposed to at least two cycles of BoNT-A injections prior to inclusion in the study and group assignment, although some patients were given a series of three treatments, occurring at 3-month intervals. At the follow-up study visit (post-Tx), patients completed the questionnaires (PHQ-9 and MIDAS) and disease severity measures (pain intensity and headache frequency over the last 4 weeks). After the follow-up visit, patients were identified via our clinical database and then assigned to one of two groups with respect to their prophylactic treatment responsivity: (1) treatment responders; or (2) treatment non-responders. Assignment to the responder group required that the patient reported at least a 50% reduction in headache frequency in the last 4 weeks and a total headache frequency <15 headache days/month at post-Tx compared to baseline (post – pre). Patients were then contacted and returned for scanning within 2-weeks of the follow-up visit. At the time of scanning all patients were determined to be interictal and not currently experiencing a migraine, which would have precluded them from being scanned. However, some patients in the non-responder group may have had low-grade background headaches. In addition, just prior to scanning, patients were asked the number of years they have been living with migraine headaches, which served as a measure of disease duration.

### BoNT-A Dosage and Administration

The injection protocol employed in this study is the standard protocol used at the Mayo Clinic Rochester since 2002. For each BoNT-A treatment series, two vials of BoNT-A (BOTOX^®^; Allergan, 2013), one containing 50 units and one containing 100 units were reconstituted and drawn into six syringes with 25 units in each 0.5 ml syringe (30 gauge × $\frac{1}{2}$ inch), for a total volume of 3 ml (150 units) were injected intramuscularly to 13 different facial and pericranial sites. Injection sites included the procerus muscle (5 units total), the left and right corrugator (10 units total; 5 units per muscle, 2 injection sites per muscle), superior frontalis (10 units total; 2.5 units in the medial and lateral aspects of right and left superior frontalis), temporalis (25 units total; two injections in the left and right temporalis; 6.25 units per injection), splenius capitis (25 units total; two injections in the left and right splenius capitis; 12.5 units per muscle), occipitalis (25 units total; two injections in the left and right occipitalis; 6.25 units per injection), and trapezius (50 units total; three injection sites per muscle; ~8.33 units per injection) muscles.

### MRI Acquisition and Preprocessing Pipeline

Image acquisition was performed with a GE Medical Systems 3 Tesla MRI scanner equipped with a 12-channel head coil. For each patient, a high-resolution, T1-weighted magnetization-prepared rapid gradient-echo sequence was acquired [slices = 176, field of view = 220 × 220, echo time = 1.74, repetition time = 2520, flip angle = 7°, resolution = 1 mm × 1 mm, slice thickness = 1 mm, no gap]. The anatomical scan was followed by a functional T2-weighted echo-planar imaging resting-state functional MRI (rs-fMRI) scan. During rs-fMRI, patients were instructed to simply relax with their eyes open (slices = 34,300 volumes, field of view = 224 mm × 224 mm, echo time = 30 ms, repetition time = 2010 ms, flip angle = 90°, resolution = 3.5 × 3.5, slice thickness = 5 mm). Diffusion tensor imaging and pseudo-continuous arterial spin labeling scans were also acquired following the rs-fMRI scan (data to be presented in a separate report). Total scan time was approximately 50 min.

Preprocessing for the surface-based morphometric analysis was performed using FreeSurfer (version 5.3.0)^1^, a semi-automated toolbox for cortical surface reconstruction and visualization (Dale et al., 1999; Fischl et al., 1999). Affine registration of the T1-weighted volume to Talairach space was performed, followed by skull stripping, white matter (WM) segmentation and tessellation of the gray/WM boundary. Visual inspection and manual correction of topological errors were carried out at each processing step. Following reconstruction of the cortical surface, brains were inflated, averaged across patients to produce a study-specific brain, and then smoothed using a 10 mm full-width at half maximum Gaussian kernel. Each hemisphere was parcellated into 34 distinct regions using the Desikan-Killany atlas (Desikan et al., 2006). A direct measure of cortical thickness was calculated using the shortest distance (mm) between the pial surface and gray-WM boundary at each point or vertex of the cortical mantle (Fischl and Dale, 2000).

For rs-fMRI data, all images were converted from DICOM to NIFTI format and then skull-stripped using FSL’s brain extraction tool. All subsequent preprocessing was performed using Statistical Parametric Mapping version 8 (SPM8; Wellcome Institute of Cognitive Neurology, London) and Matlab (R2015a). Preprocessing steps included motion correction, coregistration of anatomical image to the mean functional image, segmentation of the anatomical image into GM, WM, and cerebrospinal fluid (CSF), and normalization of anatomical and functional images to the standard Montreal Neurologic Institute (MNI) 152 brain template (voxel size = 2 mm^3^). Normalized images were then smoothed with an 8 mm isotropic full-width at half maximum Gaussian Kernel.

### Statistical Analysis Pipeline

A series of univariate analysis of variance (ANOVA) statistical tests were performed to determine whether groups (i.e., responders vs. non-responders) differed in age, intracranial volume, migraine disease characteristics (disease duration, age of disease onset) and primary and secondary endpoint measures of disease severity (headache frequency and pain intensity) assessed at baseline (pre-Tx). In addition, a series of repeated measures (RM) ANOVAs using a general linear model (GLM) approach were carried out to ascertain whether groups differed across time for clinical primary and secondary endpoints (i.e., headache frequency and pain intensity). Due to missing data for PHQ-9 and MIDAS across time points, RM ANOVA analyses were not performed for these measures.

Cortical thickness analysis for each hemisphere was conducted using FreeSurfer’s Query, Design, Estimate, Contrast (QDEC) graphical interface (version 1.5). Group comparisons were performed using a GLM design matrix with Group (responders; non-responders) specified as the fixed factor and age designated as a nuisance variable (i.e., controlled for in the analysis). Results for each analysis were overlaid onto the average inflated surface maps using QDEC. An initial cluster forming significance threshold of *p* < 0.005 with a cluster extent of 100 was used. Correction for multiple comparisons was performed using random-field-theory-based significant clusters at *p* < 0.05. Talairach coordinates for peak vertices yielding significant between-subject effects were converted to MNI space using GingerALE (version 2.3.4)^2^.

We performed a series of linear regression GLM analyses in QDEC (with age specified as a nuisance variable) to examine whether the relationship between disease duration and cortical thickness differed between groups while controlling for age. An initial cluster forming significance threshold of *p* < 0.005 and a cluster extent of 100 was used. Correction for multiple comparisons was performed using random-field-theory-based significant clusters at *p* < 0.05. Extracted data from the GLM regression analysis were graphed using scatter plots created in SPSS (version 23) to visualize relationships between groups for disease duration and cortical thickness while controlling for age.

The analysis of functional connectivity during resting-state was performed using a series of seed-to-voxel analyses with the Functional Connectivity (Conn; version 15.g) toolbox (Whitfield-Gabrieli and Nieto-Castanon, 2012). All seeds for the resting-state functional connectivity (RS-FC) analysis were generated in MarsBar^3^ using peak voxel MNI coordinates for clusters demonstrating significant group differences in cortical thickness from the surface-based analysis. The coordinates included the right primary somatosensory cortex (SI; *x* = 55.31, *y* = −14.60, *z* = 36.37), roughly corresponding to the orofacial region of the sensory homunculus, the right anterior insula (aINS; *x* = 35.84, *y* = 20.06, *z* = −0.57), the left superior temporal gyrus (STG; *x* = −54.90, *y* = −2.30, *z* = −10.82) and the left pars opercularis (ParsOp; *x* = −54.18, *y* = 9.08, *z* = −2.88). At the first-level, subjects’ warped anatomical and smoothed functional images were specified. Covariate regressors were entered into the model and included segmented WM and CSF, realignment parameters obtained during the motion correction preprocessing step in SPM8, and transient spikes identified in the fMRI time series representing motion outliers obtained from the Artifact Detection Tools software program^4^. Additionally, a denoising step was conducted using “aCompCor” method (Behzadi et al., 2007; Murphy et al., 2009; Chai et al., 2012; Whitfield-Gabrieli and Nieto-Castanon, 2012). Resting-state fMRI data were bandpass filtered (0.008–0.09 HZ) and signal associated with the six motion parameters, motion outliers, and those from WM and CSF seed regions were regressed from the rs-fMRI time series.

At the second-level, a series of voxel-wise analyses were performed and functional connectivity maps for each of the four seeds (SI, aINS, STG, ParsOp) were obtained. An initial height threshold level of *p* < 0.005 was used. Correction for multiple comparisons was accomplished with 3dClustSim using Analysis of Functional NeuroImages (AFNI^5^; Cox, 1996) software which calculated the minimum number of contiguous voxels required for a cluster to be deemed significant (2-sided threshold for *t*-tests) at an FDR of *p* < 0.05 (5000 iterations). Additionally, in order to determine whether group differences in RS-FC represented anti-correlations or simply reductions in positive connectivity, we extracted Fisher’s transformed correlation coefficients (Fisher’s r-to-z transform) using the Conn toolbox for each of the four seed regions and produced bar graphs to visually examine the nature of connectivity.

## Results

Our initial study sample consisted of 24 chronic migraineurs (18 = females; 6 = males). As previously mentioned, assignment to the responder group required a ≥50% reduction in the primary outcome measure (i.e., headache frequency) at the post-Tx relative to the pre-Tx time point (baseline). Twelve patients (females = 9, males = 3; mean age = 39.083 ± 12.01) fit this criterion and were subsequently classified as treatment responders, with the remaining 12 CM patients categorized as non-responders (females = 9, males = 3; mean age = 38.750 ± 13.98). One patient had unusable anatomical data resulting from excessive motion artifacts. Therefore, this patient’s anatomical and functional imaging data were excluded from all subsequent analyses, leaving a final sample size of 23 (11 responders and 12 non-responders).

### Demographic and Clinical Characteristics

Individual patient characteristics are displayed in Table 1. Descriptive and inferential statistics for demographic and clinical variables in treatment responders (*n* = 11; females = 8, males = 3) vs. non-responders (*n* = 12; females = 9, males = 3) are summarized in Table 2. A series of univariate GLM analyses (one-way ANOVAs) revealed no significant group differences at baseline (pre-Tx) for age, intracranial volume, disease duration or age of disease onset. In addition, there were no significant group differences at baseline for pain intensity or headache frequency. However, a RM ANOVA confirmed a significant Group (responders vs. non-responders) × Time (pre- vs. post-Tx) interaction for the primary outcome variable, headache frequency. At the post-Tx time point relative to baseline, responders showed a significant reduction (92% reduction) in headache frequency following BoNT-A treatment compared to non-responders (0% change). In addition, a RM ANOVA yielded a significant difference between groups for pre- vs. post-Tx pain intensity scores. Specifically, responders showed significant reductions (57.7%) in pain intensity due to prophylactic treatment whereas no significant change (4.2%) in pain intensity scores was observed for the non-responder group.

**Table 1:** **Patient characteristics**.

Patient	Group	Age (years)	Gender	Disease duration (years)	Pre-Tx pain intensity	Post-Tx pain intensity	Pre-Tx headache frequency	Post-Tx headache frequency
1	R	47	Female	5	−	−	28	4
2	R	32	Female	20	7	5	26	3
3	R	33	Male	20	7	7	16	1
4	R	54	Female	25	−	4	25	0
5	R	48	Female	30	8	7	16	6
6	R	50	Female	33	7	2	28	1
7	R	51	Female	33	8	0	15	0
8	R	32	Female	20	7	4	28	2
9	R	37	Female	15	8	2	25	2
10	R	18	Male	4	7	0	28	0
11	R	21	Male	6	7	0	15	2
12	NR	49	Female	15	8	8	28	28
13	NR	35	Male	25	7	7	28	28
14	NR	46	Female	2	5	5	28	20
15	NR	54	Female	30	7	9	28	20
16	NR	49	Female	26	8	6	26	26
17	NR	28	Female	19	9	8	28	28
18	NR	22	Male	7	7	6	28	28
19	NR	59	Female	44	6	6	15	24
20	NR	20	Male	10	7	7	25	29
21	NR	48	Female	3	7	6	25	28
22	NR	19	Female	12	8	8	28	28
23	NR	36	Female	9	4	4	28	28

*Abbreviations and symbols: Pre-Tx, pre-treatment; Post-Tx, post-treatment; R, responders; NR, non-responders; dashed line (−) denotes missing data*.

**Table 2:** **Descriptive and inferential statistics for responder and non-responder groups**.

Measure	Responders			Non-responders			*F* statistic	*P*
	Mean	SD	Range	Mean	SD	Range		
Age	38.45	12.39	36	38.75	13.98	40	0.003	0.958
Intracranial volume (mm^3^)	1.52 × 10^6^	2.13 × 10^5^	7.61 × 10	1.50 × 10	1.63 × 10	6.22 × 10	0.071	0.792
Disease duration	19.18	10.76	29	16.83	12.47	42	54.521	0.635
Age of disease onset	17.27	8.84	32	21.75	13.14	38	0.901	0.353
Pain intensity (pre-Tx)	7.33	0.50	1	6.92	1.38	5	0.740	0.400
Pain intensity (post-Tx)*	3.10	2.73	7	6.63	1.40	5	7.047	0.016
Headache frequency (pre-Tx)	22.73	5.85	13	26.25	3.75	13	3.013	0.097
Headache frequency (post-Tx)*	1.91	1.87	6	26.25	3.19	9	143.174	<0.001
PHQ-9 (pre-Tx)	5.00	4.08	8	11.00	5.86	16	−	−
PHQ-9 (post-Tx)	1.00	1.16	2	9.14	6.39	20	−	−
MIDAS (pre-Tx)	57.00	85.51	175	90.40	88.67	258	−	−
MIDAS (post-Tx)	13.80	17.70	36	76.78	71.35	229	−	−

Abbreviations: SD, standard deviation; pre-Tx, pre-treatment; post-Tx, post-treatment; PHQ-9, Patient Health Questionnaire, version 9; MIDAS, Migraine Disability Assessment. *Denotes F statistic and corresponding p value for repeated measures ANOVA (Group × Time) performed on pre- vs. post-Tx measures.

### Group Differences in Cortical Thickness

**Figure 1:**
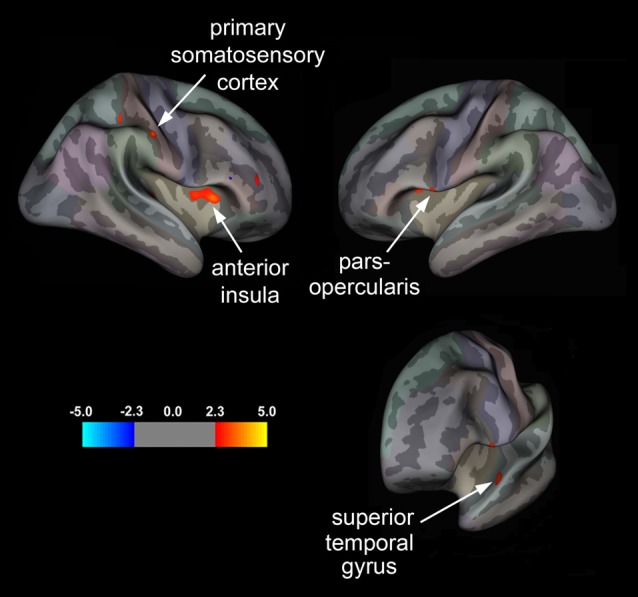
**Statistical group maps displaying differences in cortical thickness in treatment responders relative to non-responders rendered onto an average study-specific inflated cortical surface for the right and left hemisphere.** Red clusters correspond to areas displaying greater cortical thickness in responders vs. non-responders for the primary somatosensory cortex (SI) and anterior insula (aINS) in the right hemisphere and the pars opercularis (ParsOp) and superior temporal gyrus (STG) in the left hemisphere. Bottom right brain is angled to illustrate the cluster in the left STG.

Our surface-based, vertex-wise analysis revealed significant group differences in cortical thickness across multiple brain regions (Figure 1 and Table 3). In the right hemisphere, treatment responders compared to non-responders showed cortical thickening in the primary SI and aINS (Figure 1). In the left hemisphere, responders relative to non-responders showed significant cortical thickening in the STG and ParsOp (Figure 1).

**Table 3:** **Summary of brain regions showing group differences (controlling for age) in cortical thickness for responders vs. non-responders**.

Region	Side	NVtxs	Cluster size (mm^2^)	*F* value	MNI coordinates		
					*x*	*y*	*z*
SI	R	139	57.60	3.883	55.31	−14.60	36.37
aINS	R	331	135.49	3.481	35.84	20.06	−0.57
STG	L	150	86.35	3.202	−54.90	−2.30	−10.82
ParsOp	L	125	54.94	3.122	−54.18	9.08	−2.88

*Abbreviations: SI, primary somatosensory cortex; aINS, anterior insula; STG, superior temporal gyrus; ParsOp, parsopercularis; R, right; L, left; NVtxs, number of vertices; MNI, Montreal Neurological Institute*.

### Cortical Thickness and Disease Duration

**Figure 2:**
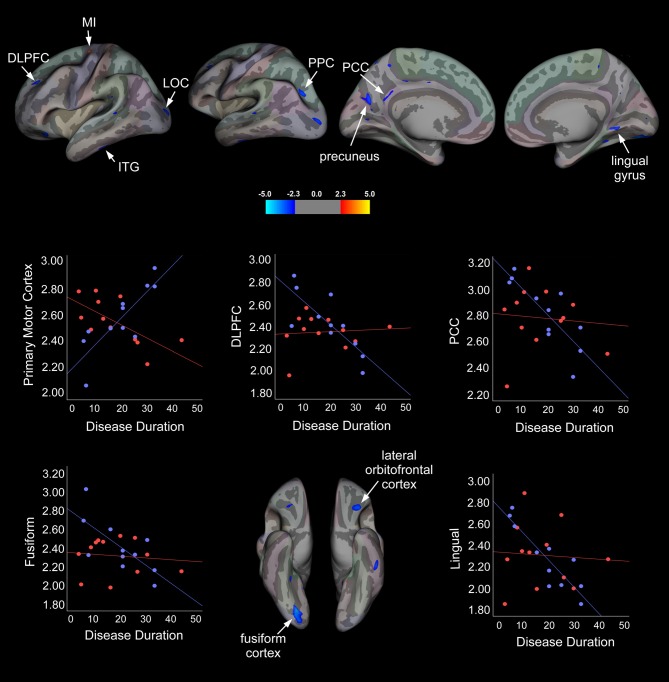
**Statistical group maps rendered onto an average study-specific inflated surface for the left and right hemispheres displaying areas showing significant group differences in slopes for associations between cortical thickness and disease duration while controlling for age.** Scatter plots illustrate differences in slopes for each group while controlling for age (blue filled circles and line correspond to treatment responders and red filled circles and line correspond to non-responders). Abbreviations: MI, Primary motor cortex; DLPFC, dorsolateral prefrontal cortex; LOC, lateral occipital cortex; ITG, inferior temporal gyrus; PPC, posterior parietal cortex; PCC, posterior cingulate cortex.

To determine whether disease duration was related to cortical thickness in responders vs. non-responders, a vertex-wise linear regression analysis with cortical thickness and disease duration specified as dependent variables and age added as a confounder was performed for each hemisphere. Summary statistics are displayed in Table 4 and statistical maps of brain areas showing group differences in cortical thickness associated with disease duration, along with corresponding scatter plots, are illustrated in Figure 2. In the left hemisphere, a strong positive association between cortical thickness in the primary motor cortex (MI) and disease duration was observed in the responder group, whereas non-responders showed a strong negative association for this region. Conversely, responders showed a moderate negative association between disease duration and cortical thickness in the left inferior temporal gyrus (ITG), while non-responders displayed a moderate positive correlation. We also observed significant group differences in cortical thickness that were related to disease duration in responders, but not for non-responders. For instance, in the treatment responder group, moderate to strong negative correlations between disease duration and cortical thickness in the left dorsolateral prefrontal cortex (DLPFC), precuneus, posterior parietal cortex (PPC), posterior cingulate cortex (PCC), and right fusiform and lingual cortical areas were identified. No significant correlations were found for cortical thickness between these areas and disease duration in the non-responder group. Interestingly, non-responders showed a strong to moderate positive association between cortical thickness in the lateral occipital cortex (LOC), but no relationship was seen in the responder group.

**Table 4:** **Summary statistics for brain regions showing associations between cortical thickness and disease duration (controlling for age) in responders vs. non-responders**.

	Side	NVtxs	Cluster size (mm^2^)	*F* value	MNI coordinates			Partial corr Responders		Partial corr Non-responders	
					*x*	*y*	*z*	*r*	*p*	*r*	*p*
MI	L	97	43.05	5.1590	−23.6	−15.5	62.1	**0.83**	**0.002**	**−0.68**	**0.016**
DLPFC	L	111	87.67	−3.4115	−24.7	36.9	35.3	**−0.80**	**0.003**	0.82	0.800
ITG	L	145	99.02	−3.1850	−55.0	−30.6	−26.4	**−0.63**	**0.039**	**0.62**	**0.032**
LOC	L	110	89.90	−3.1662	−21.1	−95.9	6.3	−0.31	0.349	**0.69**	**0.014**
OFC	R	183	130.72	−3.1535	−33.1	29.4	−12.0	−0.34	0.312	0.19	0.563
Precuneus	L	303	185.96	−3.0650	−19.9	−68.0	21.9	**−0.63**	**0.039**	0.37	0.234
PPC	L	134	65.38	−3.0402	−24.0	−70.9	26.8	**−0.90**	**<0.0001**	−0.51	0.087
PCC	L	109	42.76	−2.5911	−5.3	−51.8	23.8	**−0.82**	**0.002**	−0.09	0.776
Fusiform	R	435	377.51	−3.8774	25.1	−78.5	−7.0	**−0.71**	**0.014**	−0.12	0.717
Lingual	R	170	99.06	−3.0506	11.2	−54.6	−0.9	**−0.89**	**<0.0001**	−0.07	0.836

*Abbreviations and symbols: MI, primary motor cortex; DLPFC, dorsolateral prefrontal cortex; inferior temporal gyrus; LOC, lateral occipital cortex; OFC, orbitofrontal cortex; PPC, posterior parietal cortex; PCC, posterior cingulate cortex; R, right; L, left; NVtxs, number of vertices; MNI, Montreal Neurological Institute; corr, correlations; r, partial correlation coefficient; p, p-value. Bold values indicate correlations that reached significance*.

### Group Differences in Functional Connectivity

**Figure 3:**
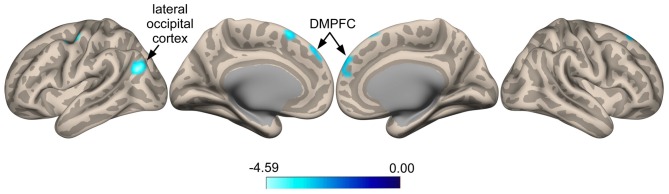
**Statistical group maps rendered onto an inflated cortical surface for the seed in the right primary somatosensory cortex (SI) showed greater anti-correlated functional connectivity with clustered voxels in the lateral occipital cortex (LOC) and dorsomedial prefrontal cortex (DMPFC) for responders vs. non-responders**.

**Figure 4:**
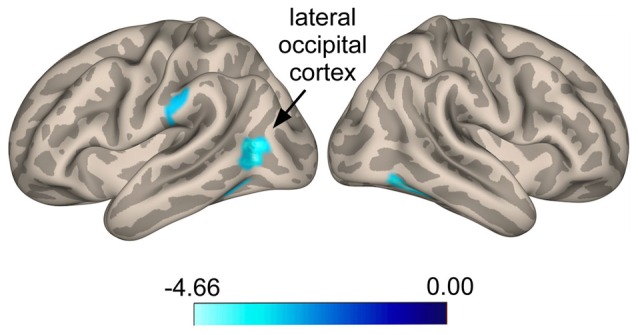
**Statistical group maps rendered onto an inflated cortical surface for the left parsopercularis (ParsOp) seed showing greater positive connectivity for voxels in the lateral occipital cortex (LOC) in responders compared to non-responders**.

Seed-to-voxel whole brain analysis revealed that responders show increased anti-correlated activity compared to non-responders between the SI seed and a cluster located in the left LOC (Ke = 361, *t*-value = 4.66, *z*-value = 3.58, cluster-level *p* = 0.022, peak-level *p* < 0.0001; *x* = −48, *y* = −68, *z* = 24), with voxels extending into the angular gyrus (Figure 3). A similar pattern was seen for a cluster in the right superior frontal gyrus (SFG; Ke = 285, *t*-value = 3.52, *z-*value = 3.08, cluster-level *p* = 0.038, peak-level *p* = 0.001; *x* = 0, *y* = 44, *z* = 48), corresponding functionally to the dorsomedial prefrontal cortex (DMPFC), with voxels for this cluster extending into the contralateral hemisphere (Figure 3). Specifically, responders compared to non-responders showed greater anti-correlated functional connectivity between the SI seed and the DMPFC. For the ParsOp seed, non-responders showed increased positive functional connectivity compared to responders for a cluster of voxels located in the left LOC (Ke = 525, *t*-value = 4.64, *z*-value = 3.81, cluster-level *p* = 0.008, peak-level *p* < 0.0001; *x* = −46, *y* = −66, *z* = 4) and extending into the inferior division of the supramarginal gyrus (SMG; Figure 4). No significant clusters were found to reach the minimum cluster threshold criteria for aINS or STG seeds.

## Discussion

The aim of this study was to identify and characterize markers of disease reversal in CM patients due to successful prophylactic treatment using multimodal neuroimaging methods. Specifically, we found that patients with CM who were responsive to the treatment intervention (responders), as evidenced by reversal from a chronic to an EM state, showed distinct structural and functional alterations in brain areas previously implicated in migraine pathophysiology, compared to CM patients that were unresponsive to treatment (non-responders). Of note, treatment responders showed increased cortical thickening in the right SI and aINS, compared to non-responders, as well as altered connectivity patterns between the SI seed and clusters in the DMPFC and LOC. In general, shorter disease durations were related to cortical thickening in numerous fronto-parietal and temporo-occipital areas in responders but not non-responders, although there were some exceptions. In addition to a decrease in the number of headache days/month, responders also showed significant reductions in headache severity (pain intensity) compared to non-responders. Taken together, our results provide further support for the plasticity of the migraine brain, and identify areas of the brain that undergo functional and structural reorganization coincident with reductions in pain severity and headache frequency in a subset of migraine patients responsive to a treatment intervention.

### Changes in Cortical Thickness in Responders vs. Non-responders

Significant group differences in cortical thickness were observed in treatment responders vs. non-responders in SI and aINS; Changes in cortical thickness were found in right SI, corresponding to the face representation of the sensory homunculus. Specifically, responders compared to non-responders showed significant cortical thickening in this area. This finding is of importance given that SI plays a key role in processing the sensory-discriminative components of pain (i.e., location and intensity). Several studies using voxel- and surface-based morphometric approaches have reported increased GM in SI in episodic migraineurs compared to healthy controls (DaSilva et al., 2007; Kim et al., 2008, 2014; Chong et al., 2016b). Data from these reports corroborate our results demonstrating SI cortical thickening in patients that reverted from a CM state to an episodic state, evidenced by a reduction in reported headache frequency. Moreover, Maleki et al. (2011) demonstrated increased cortical thickness and pain-evoked functional activation for SI in high frequency (8–14 headache days/month) compared to low frequency (<2 headache days/month) EM patients (Maleki et al., 2012). These findings support our results and suggest that cortical thickening in this area may be indicative of compensatory changes that reflect a transitionary or an in-between brain state. In addition to cortical thickening in SI, group differences were also identified in the right aINS; responders showed greater cortical thickness in this region compared to non-responders. The aINS has been implicated in autonomic and homeostatic functions (Oppenheimer et al., 1992; Critchley, 2005) as well as the processing of emotion, pain and interoceptive awareness (Craig, 2004, 2009; Critchley et al., 2004; Zaki et al., 2012; Borsook et al., 2015). Prior studies have reported a reduction in insular cortical thickness using surface-based morphometry in high-frequency episodic migraineurs compared to healthy controls (Maleki et al., 2012). Others have reported GM reductions in the insular cortex in chronic migraineurs compared to episodic (Valfré et al., 2008). We interpret that the increase in cortical thickness in SI and aINS may reflect “acute” (i.e., within months) recovery of function, and/or plastic adaptive compensatory changes in neuroconnectivity (e.g., through increased dendritic complexity, collateral sprouting, changes in synaptic strength, spine density, dendritic aborization). In support of this idea of rapid plasticity induced by “migraine relief”, relate to our findings of disease duration, which was negatively associated with cortical thickness in a number of fronto-parietal and temporo-occipital areas in the responder group. One exception was the positive association revealed in MI, with longer disease durations associated with MI cortical thickening in responders but shorter disease durations in non-responders. These rapid brain changes may relate to the inherent responsivity of patients and point to the intriguing possibility of some innate characteristic in the responder group that is predictive of BoNT-A treatment responsiveness, which is seemingly not present in the non-responders, or at least latent at the time point examined. In a seminal study, Jakubowski et al. (2006) demonstrated that patients who described their headache pain as “imploding” or “ocular” in nature showed better responsivity to BoNT-A treatment, with significant reductions in headache frequency, than those patients that reported their headache pain as “exploding”. Thus, patients’ perception of their own pain was the only factor that differentiated responders from non-responders. Although the reasons for the observed differences are beyond the scope of this study, they may reflect differences in physiology or genetics of CM patients, which may include trigeminal mechano-nociceptors response profiles, and/or genetic regulation of certain transcript factors (i.e., upregulated expression of proinflammatory genes and reduced expression of genes responsible for suppression of inflammatory response in the calvarial periosteum; Perry et al., 2016).

### Differences in Functional Connectivity in Responders vs. Non-responders

Another important finding was the altered RS-FC between responders and non-responders for regions that showed group differences in cortical thickness, notably the SI seed. Specifically, we found that responders showed increased anti-correlated RS-FC between the SI seed and voxels in the bilateral DMPFC and left LOC. The DMPFC is thought to be involved in higher-order executive functions including self-focused attention, social cognition (ToM, empathy), introspection and appraisal of negative emotions (de Wit et al., 2006, 2009;D’Argembeau et al., 2007; Etkin et al., 2011; Bzdok et al., 2012; Schilbach et al., 2012). This area is also considered a key node of the default mode network (DMN; Raichle et al., 2001). Abnormal MPFC-DMN functional connectivity patterns have been reported in patients with various chronic pain conditions, including temporomandibular disorder (Kucyi et al., 2014) and migraine (Hubbard et al., 2014). In contrast, the LOC is an extrastriatal area known to play an important role in the perception and recognition of object and faces as well as body parts (Malach et al., 1995; Kourtzi and Kanwisher, 2000; Grill-Spector et al., 2001; Taylor and Downing, 2011). Although relatively rare, it is interesting to note that complex visual and somesthetic disturbances do occur in some patients with migraine either during the ictal or interictal phase, clinically referred to as “Lilliputian hallucinations” (Lippman, 1952; Podoll and Robinson, 2000, 2002; Dooley et al., 2014; Liu et al., 2014). There is some neuroimaging evidence linking these types of visual and somesthetic disturbances to abnormal functioning of cortical regions that include the parietal-temporo-occipital junction (Kuo et al., 1998; Brumm et al., 2010). It remains to be seen whether the increase in anti-correlated activity in responders relative to non-responders reflects a decoupling of sensorimotor networks involved in nociceptive signal transmission and sensory-discriminative components of pain and networks implicated in higher-level visuospatial and self-referential cognitive control processes.

### Specificity of Treatment Effect

As noted in the introduction, the treatment intervention, while reportedly useful in patients with CM, is not effective in all patients. This may be a result of a number of factors including but not limited to individual differences in disease severity, genetics, physiology, environmental variables, medication usage, ethnicity, sex, age, comorbid pain conditions, heterogeneity of the disease and/or sequelae. Groups were matched on sex, ethnicity and age with the latter variable controlled for in our analysis, therefore it is unlikely that any of these factors explain the observed group differences in treatment responsivity. Furthermore, prior to prophylactic treatment, groups did not differ in migraine characteristics (disease duration, age of disease onset) or disease severity measures (pain intensity, headache frequency). However, it remains a possibility that successful treatment in the responder group may be due to placebo or the premorbid brain state. This study was not conducted to measure placebo effects nor did we evaluate pre-treatment state and so we cannot comment on whether the brain changes observed related to the aforementioned factors, nor can we rule out these possibilities. Regardless, the potential effector, drug or placebo, or even the natural course of the disease is less relevant than the specific differences we observed. That is, the specific treatment effector is less relevant than the outcome *per se*. However, the plasticity of the brain in CM seems likely and is supported by prior imaging studies that have evaluated the effects of opioid detoxification on reversal of CM to EM (Riederer et al., 2013).

### Limitations and Study Caveats

Limitations of this study include the small sample size, the retrospective study design and the lack of headache diaries documenting the treatment effect, given that patient retrospective self-reports are unreliable and may be prone to memory related biases. As noted above, it is not clear from the current investigation the extent to which treatment responsiveness was due to placebo or prophylactic treatment effects, or the premorbid brain state. Further studies are needed in a larger sample with a placebo arm using a prospective, longitudinal study design, to begin to disentangle the efficacy of the treatment intervention (i.e., whether drug or placebo) and its contributions to the observed morphological and functional brain changes and whether these changes represent reliable markers of migraine chronification and reversal. A promising approach for future studies may be to explore the use of machine learning to differentiate responders vs. non-responders using the functional and/or structural brain changes identified here employing similar methods developed by López et al. (2013).

### Conclusion

The migraine brain even in the migraine free state is structurally and functionally altered and “functions” abnormally. Despite all of the findings to date, however, no reliable biomarker exists for what appears to be an abnormal brain state and how the disease progression further alters the brain. Having such a biomarker would allow for: (a) a better understanding of the disease; (b) objective measures of the interictal state and may provide an index of transition from acute migraine to chronic daily headache; and (c) the ability to monitor potential benefits of therapeutic interventions including clinical trials where beneficial changes may take longer than current trials may elucidate. Moreover, a better understanding of the specific pathways or structures involved in the disease progression (or regression) may provide a unique signature for the underlying dysfunction leading to migraine in the first place. Therefore, these specificities may impart a newfound understanding of the detrimental effects of increased frequency and duration of repeated migraine headaches on the brain, and uncover the potential underlying mechanisms that trigger the progression of the disease to more severe or chronic stages. The acquirement of this knowledge could eventually help with the development of better therapeutic or preventive interventions.

Our results may provide novel insights into the brain changes that are coincident with the “resistant” compared with the “responsive” phenotype to the same prophylactic treatment. As with many treatment interventions for CM, randomized control studies frequently do not evaluate subgroups and the overall response rates may be tempered by patient response profiles. In the present study, we observed significant differences in brain structure and function in responders vs. non-responders, whether or not these changes can be ascribed to the premorbid brain state or the effect of the treatment in responders is an avenue for future research. This type of approach may provide a unique strategy for understanding brain systems in migraineurs that confer or predict responders vs. non-responders.

## Author Contributions

DB, FMC, LB and RB: designed the study; JHS, JMD, RMS, DFB, KW and FMC: performed neurological exams and collected data; CSH and LB performed statistical analyses; CSH, DB, LB: RB and FMC: prepared the manuscript; all authors contributed to the manuscript revisions.

## Funding

This study was supported by a grant from the National Headache Foundation (DB and FMC) and National Institutes of Health (NIH) K24NS064050 (DB) and Mayo Clinic Small Projects Grant (to FMC).

## Conflict of Interest Statement

The authors declare that the research was conducted in the absence of any commercial or financial relationships that could be construed as a potential conflict of interest.

## Footnotes

^1^http://surfer.nmr.mgh.harvard.edu

^2^www.brainmap.org/ale/

^3^http://marsbar.sourforge.net

^4^www.nitrc.org/projects/artifact_detect/

^5^http://afni.nimh.nih.gov/afni/
